# A pilot study of the effect of spironolactone therapy on exercise capacity and endothelial dysfunction in pulmonary arterial hypertension: study protocol for a randomized controlled trial

**DOI:** 10.1186/1745-6215-14-91

**Published:** 2013-04-02

**Authors:** Jason M Elinoff, J Eduardo Rame, Paul R Forfia, Mary K Hall, Junfeng Sun, Ahmed M Gharib, Khaled Abd-Elmoniem, Grace Graninger, Bonnie Harper, Robert L Danner, Michael A Solomon

**Affiliations:** 1 National Institutes of Health, Critical Care Medicine Department, Mark O. Hatfield Clinical Research Center, 10 Center Drive, Building 10, Room 2C145, Bethesda, MD 20892, USA; 2 Cardiovascular Medicine Division, Department of Medicine, Hospital of the University of Pennsylvania, Philadelphia, PA 19104, USA; 3 National Institutes of Health, Biomedical and Metabolic Imaging Branch, National Institute of Diabetes and Digestive and Kidney Diseases, 10 Center Drive, MSC 1662, Bethesda, MD 20892, USA; 4 National Institutes of Health, Cardiovascular and Pulmonary Branch, National Heart, Lung and Blood Institute, 10 Center Drive, Bethesda, MD 20892, USA

**Keywords:** Magnetic resonance imaging, Microarray, Mineralocorticoid receptor antagonist, Neurohormonal axis, Pulmonary arterial hypertension, Right ventricular function, Vascular inflammation

## Abstract

**Background:**

Pulmonary arterial hypertension is a rare disorder associated with poor survival. Endothelial dysfunction plays a central role in the pathogenesis and progression of pulmonary arterial hypertension. Inflammation appears to drive this dysfunctional endothelial phenotype, propagating cycles of injury and repair in genetically susceptible patients with idiopathic and disease-associated pulmonary arterial hypertension. Therapy targeting pulmonary vascular inflammation to interrupt cycles of injury and repair and thereby delay or prevent right ventricular failure and death has not been tested. Spironolactone, a mineralocorticoid and androgen receptor antagonist, has been shown to improve endothelial function and reduce inflammation. Current management of patients with pulmonary arterial hypertension and symptoms of right heart failure includes use of mineralocorticoid receptor antagonists for their diuretic and natriuretic effects. We hypothesize that initiating spironolactone therapy at an earlier stage of disease in patients with pulmonary arterial hypertension could provide additional benefits through anti-inflammatory effects and improvements in pulmonary vascular function.

**Methods/Design:**

Seventy patients with pulmonary arterial hypertension without clinical evidence of right ventricular failure will be enrolled in a randomized, double-blinded, placebo-controlled trial to investigate the effect of early treatment with spironolactone on exercise capacity, clinical worsening and vascular inflammation *in vivo*. Our primary endpoint is change in placebo-corrected 6-minute walk distance at 24 weeks and the incidence of clinical worsening in the spironolactone group compared to placebo. At a two-sided alpha level of 0.05, we will have at least 84% power to detect an effect size (group mean difference divided by standard deviation) of 0.9 for the difference in the change of 6-minute walk distance from baseline between the two groups. Secondary endpoints include the effect of spironolactone on the change in placebo-corrected maximal oxygen consumption; plasma markers of vascular inflammation and peripheral blood mononuclear cell gene expression profiles; sympathetic nervous system activation, renin-angiotensin-aldosterone system activation and sex hormone metabolism; and right ventricular structure and function using echocardiography and novel high-resolution magnetic resonance imaging-based techniques. Safety and tolerability of spironolactone will be assessed with periodic monitoring for hyperkalemia and renal insufficiency as well as the incidence of drug discontinuation for untoward effects.

**Trial registration:**

ClinicalTrials.gov: NCT01712620

## Background

### Epidemiology and natural history

The vascular injury-induced forms of pulmonary hypertension (PH), collectively referred to as pulmonary arterial hypertension (PAH), are distinct from other causes of PH such as left-sided heart failure, parenchymal lung disease with hypoxemia and chronic thromboembolic disease (Table 1) [1]. Idiopathic pulmonary arterial hypertension (IPAH) is an unexplained form of PAH where the triggering insult to the endothelium is unclear. Several diseases may manifest PAH (referred to as disease-associated PAH) that is histopathologically identical to IPAH. The underlying process responsible for these primary diseases are thought to injure the pulmonary endothelium and ultimately lead to the development of PAH (Table 1.) [1].

**Table 1:** Clinical classification of pulmonary hypertension

**Group**	**Etiology**
1	Pulmonary arterial hypertension
1.1	Idiopathic pulmonary arterial hypertension
1.2	Heritable
1.2.1	Bone morphogenetic protein receptor 2
1.2.2	Activin receptor-like kinase type 1, endoglin
1.2.3	Unknown
1.3	Drug- and toxin-induced
1.4	Associated with
1.4.1	Connective tissue diseases
1.4.2	Human immunodeficiency virus (HIV) infection
1.4.3	Portal hypertension
1.4.4	Congenital heart diseases
1.4.5	Schistosomiasis
1.4.6	Chronic hemolytic anemia
1.5	Persistent pulmonary hypertension of the newborn
1’	Pulmonary veno-occlusive disease and/or pulmonary capillary hemangiomatosis
2	Pulmonary hypertension owing to left heart disease
3	Pulmonary hypertension owing to lung diseases and/or hypoxia
4	Chronic thromboembolic pulmonary hypertension
5	Pulmonary hypertension with unclear multifactorial mechanisms

PAH is two to four times more common in woman with a mean age at diagnosis of 50.1 ±14.4 years, but it may occur at any age [2]. Prior to recent advances in treatment, the median survival for all patients in the National Institutes of Health (NIH) registry was 2.8 years; the 1-, 3- and 5-year survivals were 68%, 48% and 34%, respectively [3]. With the advent of epoprostenol therapy, the 1-, 3- and 5-year survivals for patients with IPAH has improved to 87%, 63% and 54%, respectively [4], but mortality remains unacceptably high. Therefore, therapeutic approaches that modify the natural history of IPAH and disease-associated PAH have become a major focus.

### Pathophysiology of pulmonary arterial hypertension and the role of endothelial inflammation

The initial mechanisms responsible for the development of IPAH and other forms of PAH remain incompletely understood. Independent of the inciting event, endothelial dysfunction appears to play a central role both in the pathogenesis and progression of PAH [5,6]. Inflammation has been implicated as a driver of this dysfunctional endothelial phenotype, propagating cycles of injury and repair in genetically susceptible patients with IPAH and patients with disease-associated PAH [7-9]. Histologic specimens from patients with IPAH reveal the presence of inflammatory cells, including macrophages and T- and B-lymphocytes, within classic plexiform lesions that are the hallmark of PAH [7,10]. Independent of the effects on vascular remodeling, endothelial inflammation may directly impact right ventricular (RV) function [11], possibly explaining the imperfect relationship between worsening RV function despite improvements in pulmonary vascular resistance in patients on standard therapy for PAH [12].

### Nuclear receptors and inflammation

Despite strong epidemiological evidence demonstrating a female predominance in PAH, the underlying mechanisms for this gender inequality are unclear [13]. In recent animal models of PAH that simulate features of the human disease such as occlusive or angioproliferative pulmonary vascular beds with formation of plexiform lesions and endothelial disruption, female rats develop more severe PAH than males [14]. The importance of sex hormone signaling in PAH suggests a potential therapeutic role for nuclear receptor (NR) ligands and their cognate receptors. The steroid hormone receptors, including estrogen receptor-α and -β, androgen receptor, glucocorticoid receptor, mineralocorticoid receptor (MR) and progesterone receptor, are a subgroup of the NR family of transcription factors. Our laboratory is interested in investigating anti-inflammatory NR pathways that specifically target the endothelium.

MR antagonists have been widely used in patients with left-sided heart failure or left ventricular (LV) dysfunction post-myocardial infarction [15,16]. The clinical benefit of MR antagonists in these patients has been linked to poorly characterized anti-inflammatory properties and less so to their better understood diuretic or natriuretic effects [17,18]. Spironolactone, a combined MR and androgen receptor antagonist, improves endothelial dysfunction in patients with left-sided heart failure [19-21], rheumatoid arthritis [22], and in women with polycystic ovarian syndrome [23], suggesting a possible mechanism for its clinical efficacy in these patient populations. One group has suggested that spironolactone interferes with NFκB signaling in human monocytes in a non-MR dependent manner, but the molecular basis for this conclusion has not been further clarified [24,25]. In a recent study utilizing two different animal models of PH, MR antagonists demonstrated beneficial effects on aldosterone-induced pulmonary vascular remodeling, RV hypertrophy and oxidant stress [26]. Current management of patients with severe PAH and New York Heart Association/World Health Organization (NYHA/WHO) class IV symptoms includes use of MR antagonists once clinical right heart failure has developed for their diuretic and natriuretic effects [27]. Initiating therapy with spironolactone at an earlier stage of disease in patients with PAH is a novel approach to treatment and could provide benefits through anti-inflammatory mechanisms and improvements in endothelial function that might alter the natural history of PAH. To date, however, there have been no randomized clinical trials examining the safety and efficacy of MR antagonist therapy in PAH without right heart failure. *In vitro* data from our laboratory demonstrate that spironolactone suppresses NFκB-mediated inflammatory signaling in human endothelial cells (unpublished results). We are actively investigating the molecular mechanisms that mediate the anti-inflammatory activity of spironolactone and the relative contributions of MR, androgen receptor and progesterone receptor, as well as NR-independent effects.

## Methods/Design

### Objectives

Patients with PAH (that is, Group 1 PH, Table 1) without RV failure on either no medical therapy or stable medical therapy for at least 4 weeks will be recruited to the NIH Clinical Center for a randomized, double-blinded, placebo-controlled study of early treatment with spironolactone to investigate the effect of treatment on exercise capacity, clinical worsening and vascular inflammation *in vivo*. In addition, safety and tolerability will be assessed with periodic monitoring for hyperkalemia, renal insufficiency and overall incidence of study drug intolerance relative to placebo.

### Overview of study design and study population

This study adheres to the principles of the Declaration of Helsinki and has been approved by the NIH Clinical Center Deputy Ethics Counselor and the National Heart, Lung, and Blood Institute (NHLBI) Institutional Review Board (12-CC-0211). Written informed consent will be obtained from all participants at the NIH Clinical Center prior to undergoing any testing or treatment described in this protocol. Most eligible patients will be referred from Pulmonary Hypertension Specialty Clinics. At the time of enrollment, each patient will be asked to identify a primary responsible licensed practitioner who will be notified of the patient’s enrollment and made aware of the potential development of adverse study drug effects (such as hyperkalemia and renal insufficiency) during the course of the trial. If a patient does not have a physician with expertise in PH, then they will either be referred to a physician with such expertise or they will be provided ‘standard of care’ therapy at the NIH. Eligibility will be based on information obtained from our Natural History Study and from any additional information provided by the referring physician. Participants must be at least 18 years of age and must be able to provide informed, written consent for participation in this study. There is no exclusion based on race or gender. Women of childbearing potential must agree to use adequate contraception prior to and for the duration of study participation.

Eligible individuals are patients with PAH (that is, Group 1 PH, Table 1) without clinical evidence of RV failure receiving either no medical therapy or stable medical therapy for at least the past 4 weeks (defined as no new PAH-specific therapy, no change in the dose of current PAH-specific therapy and no change in NYHA/WHO functional classification). The following parameters on right heart catheterization are required to meet the hemodynamic definition of PAH: mean pulmonary artery pressure of >25 mmHg at rest, pulmonary capillary wedge pressure of ≤15 mmHg (or LV end-diastolic pressure ≤12 mmHg) and pulmonary vascular resistance of >3 Wood units (240 dyn ⋅s ⋅cm^-5^). If clinically indicated at the time of enrollment, then a right heart catheterization will be performed at the NIH Clinical Center upon study entry. Exclusion criteria are as follows:

1. Patients with WHO Group 1 PH and evidence of right heart failure as defined by:

a. NYHA/WHO class IV symptoms

AND

b. Echocardiographic evidence of severe RV dysfunction

AND

c. Clinical signs which may include, but are not limited to elevated jugular venous pressure, ascites, and lower extremity edema

2. Patients with WHO Group 1 PH and a prior diagnosis of cirrhosis with portal hypertension as evidenced by a history of ascites, hepatic encephalopathy and/or varices prior to enrollment.

3. Patients with WHO Group 1 PH and evidence of active infection. (Patients with HIV infection and two consecutive viral loads of <500 on their most recent determinations in the past 12 months will be considered to have inactive infection.)

4. Patients with WHO Group 1 PH who have taken spironolactone or eplerenone within the last 30 days.

5. Known or suspected allergy to spironolactone.

6. Pregnant or breastfeeding women.

7. Age <18 years.

8. Inability to provide informed written consent for participation in the study.

9. Chronic kidney disease defined as estimated glomerular filtration rate (eGFR) of <50 mL/min/1.73 m^2^ of body surface area. (eGFR will be calculated using the isotope dilution mass spectrometry-traceable Modification of Diet in Renal Disease Study equation and corrected for body surface area as follows: eGFR = 175 × (serum creatinine in mg/dL)^-1.154^ × (age in years)^-0.203^ × [1.212 if African-American] × [0.742 if female].)

10.  Serum potassium at the time of enrollment of >5 mEq/L or current use of potassium-sparing diuretics, angiotensin-converting enzyme (ACE) inhibitors, angiotensin II receptor blockers or drospirenone-containing oral contraceptives.

All participants will undergo baseline testing under our Natural History protocol and serial (12 and 24 weeks) testing under this protocol according to the schedules outlined in Additional file 1: Figure S1 (all time points are approximations, ±7 days). Following each interval assessment period (baseline, 12 and 24 weeks), patients will be discharged home under the care of their referring and/or primary physicians. During the study, participants are permitted to have other medications, including PAH-specific therapies, adjusted by their primary physician as clinically necessary.

### Study endpoints

Our primary endpoint is the effect of spironolactone therapy on the change in placebo-corrected 6-minute walk distance at 24 weeks relative to baseline values and the incidence of clinical worsening in the spironolactone group compared to placebo. Clinical worsening will be defined as the addition of a new PAH-specific medication (a prostacyclin, an endothelin-1 receptor antagonist or a phosphodiesterase-5 inhibitor), dose or route escalation (that is inhaled prostacyclin changed to intravenous prostacyclin) of a current PAH-specific medication, development of right heart failure requiring hospitalization or an escalation in outpatient diuretic therapy (that is, initiation of a new diuretic or a dose escalation of current diuretic therapy), transplantation, or death.

Secondary endpoints include effect of spironolactone therapy on exercise capacity, as measured by the change in placebo-corrected VO_2_ maximum at 24 weeks relative to baseline values; effect of spironolactone therapy on plasma markers of vascular inflammation (see Table 2) and peripheral blood mononuclear cell (PBMC) gene expression profiles, comparing changes from baseline between the treatment and placebo groups; effect of spironolactone therapy on sympathetic nervous system activation, renin-angiotensin-aldosterone system activation and sex hormone metabolism, comparing changes from baseline between the treatment and control groups (see Table 2); effect of spironolactone therapy on RV function as assessed by echocardiography, magnetic resonance imaging (MRI) and N-terminal pro-brain natriuretic peptide (NT-proBNP), comparing changes from baseline between the treatment and placebo groups; and safety and tolerability of spironolactone therapy as determined by the rate of study drug discontinuation due to hyperkalemia (serum potassium ≥5.3 mEq/L), renal insufficiency (serum creatinine rise >50% of baseline), or other side effects such as breast pain and gynecomastia compared to placebo.

**Table 2:** Biomarker assessment

**Markers of endothelial dysfunction**	**Neurohormonal axis**
**Adhesion molecules**	**Sympathetic activation**
sICAM-1, sVCAM-1, sE-selectin	Epinephrine, norepinephrine, arginine vasopressin
**Markers of activated coagulation**	**Renin-angiotensin-aldosterone system activation**
von Willebrand factor, thrombomodulin, sP-selectin	Renin activity, aldosterone, angiotensin II, NT-proBNP
**Cytokines and chemokines**	**Sex hormone metabolism**
TNFα, IL-1β, IL-6, IL-7, IL-8, MCP-1, sCD40L, IL1RL1	Androstenedione, dehydroepiandrosterone sulfate, free testosterone, estradiol, 2-methoxyestradiol, urine 2-hydroxyestradiol:16α-hydroxyestrone ratio

Abbreviations: IL-1β, interleukin-1 beta; IL1RL1, interleukin-1 receptor-like 1; IL-6, interleukin-6; IL-7, interleukin-7; IL-8, interleukin-8; MCP-1, monocyte chemotactic protein-1; NT-proBNP, N-terminal pro-brain natriuretic peptide; sCD40L, soluble CD40 ligand; sICAM-1, soluble intercellular adhesion molecule 1; sVCAM-1, soluble vascular cell adhesion molecule 1; TNFα, tumor necrosis factor alpha.

### Treatment delivery

**Figure 1:**
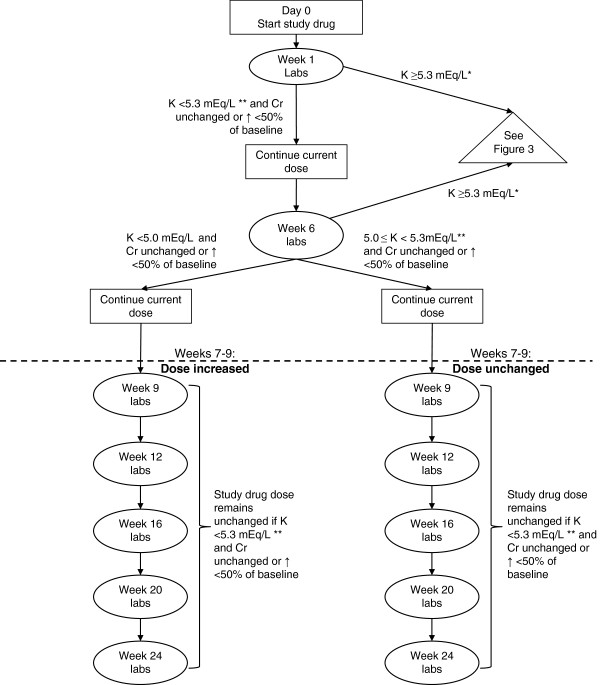
**Serum potassium and creatinine monitoring schedule.** *Participants with potassium level ≥5.3 at week 1 will not be eligible for dose increase at 8 weeks. **Throughout the study, participants with potassium level >5.0 will undergo dietary counseling regarding a low potassim diet. Cr, creatinine; K, potassium; labs, laboratory tests.

The total duration of the treatment period will be 24 weeks. Treatment assignment will be double-blinded. Randomization will be computer-generated and participants will be stratified according to the cause of PAH (idiopathic, heritable, and drug or toxin-induced PAH versus disease-associated PAH) and NYHA/WHO classification (I or II versus III; see Table 3). Participants meeting eligibility criteria for enrollment will be randomized to either 25 mg of spironolactone or matching placebo once daily. The NIH Clinical Center Pharmaceutical Development Section will be compounding and monitoring study drug stability. Four different capsules will be prepared: spironolactone 25 mg and matching placebo, and spironolactone 50 mg and matching placebo. If the 25 mg dose of study drug is well tolerated, (that is, potassium remains <5.0 mEq/L and serum creatinine does not increase >50% from baseline at study entry) then the dose will be increased to 50 mg daily following week 6 laboratory monitoring (Figure 1).

**Table 3:** New York Heart Association/World Health Organization classification

**Class**	**Symptoms**
I	Patients with pulmonary hypertension but without resulting limitation of physical activity. Ordinary physical activity does not cause undue dyspnea or fatigue, chest pain or near-syncope.
II	Patients with pulmonary hypertension resulting in slight limitation of physical activity. They are comfortable at rest. Ordinary physical activity causes undue dyspnea or fatigue, chest pain or near-syncope.
III	Patients with pulmonary hypertension resulting in marked limitation of physical activity. They are comfortable at rest. Less than ordinary activity causes undue dyspnea or fatigue, chest pain or near-syncope.
IV	Patients with pulmonary hypertension with inability to carry out any physical activity without symptoms. These patients manifest signs of right heart failure. Dyspnea and/or fatigue may even be present at rest. Discomfort is increased by any physical activity.

All participants will receive new study drug in the mail between weeks 7 and 9 of the study. Participants who are not eligible for the dose increase (Figure 1) will also receive new study drug but the dose will be unchanged. The 25 mg and 50 mg dosage formulations will each be a different colored capsule so that the participants and providers can easily discern them. Two different color-matched placebo capsules will be formulated as well. Study coordinators will contact participants and instruct them to begin the new study medication at the appropriate time. Participants will also be instructed to mail their old study medication bottles back to the NIH Clinical Center to avoid confusion with the new study drug.

All participants randomized to either spironolactone or placebo will be counseled on avoiding foods high in potassium and avoiding concurrent use of nonsteroidal anti-inflammatory drugs (NSAIDs). The NIH Clinical Center Pharmacy conducted a formal drug-drug interaction evaluation using Micromedex^®^ (Truven Health Analytics Inc., Ann Arbor, MI, USA), Lexi-Interact™ (Lexi-Comp Inc., Hudson, OH, USA), the package insert for spironolactone, and a focused literature search. No significant interactions were found for spironolactone or any of the PAH-specific drug classes or for HIV antiretroviral therapy. However, phosphodiesterase-5 inhibitors and prostacyclin analogs may enhance the hypotensive effect of any class of antihypertensive medications.

Patients taking potassium supplements at the time of screening will be eligible for enrollment if the primary treating physician and the study investigators believe that it is clinically safe to decrease the current dose or stop the supplement depending on the clinical situation. Potassium will be monitored frequently during this protocol and, if necessary, potassium supplementation can be resumed based on standard clinical practice.

### Protocol procedures

#### Modified New York Heart Association/World Health Organization functional classification of pulmonary hypertension

The modified NYHA/WHO functional classification provides a simple way of classifying the extent of functional impairment in patients with PH [28]. It places patients in one of four categories based on how much they are limited during ordinary physical activity (Table 3).

#### Six-minute walk test

The 6-minute walk test has been used successfully to measure the effectiveness of therapy and risk stratification in heart failure and PH. This test is indicative of an individual’s ability to perform activities of daily living [29,30]. The 6-minute walk test has demonstrated reproducibility in a spectrum of patients with chronic cardiopulmonary disease [30-34]. The coefficient of variance has been reported at <10% [30,32]. As a result, the 6-minute walk test has been accepted by regulatory agencies and is most commonly used as a primary endpoint in randomized clinical trials of PAH-specific therapy [33]. Reference equations for estimating 6-minute walk distance in healthy men and women are based on height, age and weight. In PAH, the distance walked significantly decreases with worsening NYHA/WHO functional class and has been independently related to mortality in PAH by multivariate analysis [35]. The 6-minute walk test will be performed in accordance with standard practice. Each participant will walk along a measured pathway with pulse oximetry and heart rate monitoring. Blood pressure, oxygen saturation, heart rate and a modified Borg dyspnea index will be recorded at baseline, upon completion of the test, and during recovery.

#### Cardiopulmonary exercise testing

Parameters obtained during cardiopulmonary exercise testing, peak oxygen uptake (peak VO_2_) and the slope of ventilation to carbon dioxide production during exercise (VE/VCO_2_) have been well established as powerful prognostic indicators in chronic heart failure. In patients with IPAH, cardiopulmonary exercise testing demonstrated that strong predictors of impaired survival were low peak VO_2_ and low systolic blood pressure at peak exercise [36]. Patients with peak VO_2_ ≤10.4 ml/kg/min and peak systolic blood pressure ≤120 mmHg had poor survival rates at 12 months (23%) compared to patients with one or neither of these risk factors (79% and 97% respectively).

All participants will undergo symptom-limited treadmill exercise testing using a modified Naughton protocol (Table 4). This protocol is designed to gently increase work by limiting each stage increase to approximately one metabolic equivalent (3.5 ml/kg/min VO_2_). The following will be recorded: exercise duration; heart rate, systemic blood pressure and pulse oximetry at the start of the exercise, at the end of each 2-minute stage of the exercise protocol, and immediately after cessation of exercise; and oxygen consumption and carbon dioxide production using breath-by-breath analysis of expired gas (Sensor Medics Horizons MMC, Anaheim, CA, USA) with each 15 seconds of data averaged. Any individual unable to complete the cardiopulmonary exercise testing but otherwise willing to participate in the protocol will remain eligible and their data will be included in any final analyses of the portions of the protocol that they completed.

**Table 4:** Modified naughton protocol for exercise testing

**Stage**	**Time** (**minutes**)	**MPH**	% **Incline**	**METS**
0 (rest/recovery)	1:00	1.0	0.0	1.8
1	2:00	2.0	0.0	2.5
2	2:00	2.0	3.5	3.4
3	2:00	2.0	7.0	4.4
4	2:00	2.0	10.5	5.4
5	2:00	2.0	14.0	6.4
6	2:00	2.0	17.5	7.3
7	2:00	2.6	14.0	8.0
8	2:00	3.0	14.0	9.1
9	2:00	3.4	14.0	10.2
10	2:00	3.7	14.0	11.0

MET, metabolic equivalent (3.5 ml/kg/min VO_2_); MPH, miles per hour.

#### Transthoracic echocardiogram

Echocardiograms will be performed according to standard American Society of Echocardiography criteria using commercially available ultrasound machines. Images will be recorded and saved on an online server. If technically possible, multiple views will be recorded including the parasternal long- and short-axis views, the apical two- and four-chamber views, and the subcostal views. Cardiac chamber sizes will be assessed from multiple views. RV outflow tract flow will be assessed with the sample volume positioned immediately below the pulmonic valve in the parasternal short-axis view with pulse wave and continuous wave Doppler. Analysis of the pulsed wave Doppler signal in the RV outflow tract will allow for measurement of the velocity time integral (surrogate of stroke volume), time to peak velocity (acceleration time) and for categorization of the basic morphology of the Doppler signal (no notch, late systolic notch, mid-systolic notch) [37]. The RV diastolic diameter will be measured 1 cm apical of the tricuspid annulus from the apical four-chamber view. The LV diastolic diameter will also be assessed 1 cm apical of the mitral annulus from the apical four-chamber view. RV end-diastolic dimension can thus be expressed as an absolute value and in relation to LV end-diastolic dimension (RV:LV dimension ratio). Atrial areas will be measured in the same view by planimetry at end-systole. Color Doppler will be used to assess the degree of tricuspid regurgitation in multiple views. The highest right atrial to right ventricular gradient from continuous wave Doppler will be used as an estimate of RV systolic pressure. The spectral jets of tricuspid and mitral regurgitation with continuous wave Doppler will be used for measurement of dP/dt. If technically feasible, subcostal views will be used to assess inferior vena cava size and response to respiratory maneuvers. Tricuspid annular plane systolic excursion will be measured using either M-mode imaging or two-dimensional imaging from the apical four-chamber view [38]. Tissue Doppler imaging will be used for assessment of LV and RV systolic and diastolic performance. Tissue velocity data will be used to derive strain and strain rate measurements for assessment of ventricular systolic and diastolic performance.

#### Magnetic resonance imaging

Assessment of RV structural and functional remodeling remains a fundamental aspect of PAH diagnosis and follow-up [39]. Echocardiography is currently the most commonly used diagnostic tool for these assessments, however, it suffers from many limitations such as limited viewing angles, low signal-to-noise ratio and a low frame rate [40]. Moreover, only longitudinal contraction has been investigated, which represents strain in only one direction and is prone to inaccurate measurements of the RV due to its complex crescent shape [40,41]. RV imaging using MRI has recently gained interest because of the quality and quantity of data obtained with this technique. Unfortunately, assessing changes in global RV structure as well as local contractility still pose difficulties for currently used MRI techniques. Some of these limitations include low functional resolution and prohibitively long image acquisition times in order to obtain three-dimensional contractility data. Our collaborators have recently developed fast MRI techniques for quantification of three-dimensional myocardial strain from single slices [42,43], and for high functional resolution imaging. These new techniques address previous limitations and may allow for a more accurate assessment of RV structural and functional changes and how they evolve with disease progression.

In addition to developing more sophisticated MRI techniques of assessing the RV in patients with PAH, there may also be an important role for using MRI to follow pulmonary artery endothelial function over time and in response to therapy. Currently, much of the interpretation for the role of endothelial function in underlying disease states is based on studies of either peripheral vessel dynamic vasomotor function, which only modestly correlates with that of the main arteries [44-46], or using invasive angiography with Doppler flow measures. MRI is another method of non-invasively assessing arterial cross-sectional area and flow velocity, however, it has been rarely used for endothelial-dependent vasomotor responses [47,48]. Utilizing newer MRI methods, it is now possible to quantify pulmonary arterial vasoreactivity as a surrogate for pulmonary artery endothelial function.

The MRI sequence protocol includes global measures, such as end-diastolic and end-systolic RV/LV volume and RV/LV ejection fractions; local measures, such as RV systolic and diastolic peak longitudinal strain and strain rates and three-dimensional regional contractility (strain) analysis; and vasomotor responses. To assess endothelial-dependent vasomotor responses, each participant will be asked to perform the hand-grip exercise while arterial dimensions and blood flow measurements are obtained [49,50]. Magnetic resonance data will be collected before, during and after hand-grip exercise. The vascular beds of interest include, but are not limited to, the coronary, brachial and pulmonary arteries. Exclusion criteria for MRI and gadolinium are listed in Table 5. Any individual unable to tolerate some or all of the MRI sequences included in the study but otherwise willing to participate in the protocol will remain eligible and their data will be included in any final analyses of the portions of the protocol that they completed.

**Table 5:** Magnetic resonance imaging and gadolinium contrast injection exclusion criteria

**These contraindications include but are not limited to the following devices or conditions:**	
1.	Implanted cardiac pacemaker or defibrillator
2.	Cochlear Implants
3.	Ocular foreign body (for example, metal shavings)
4.	Embedded shrapnel fragments
5.	Central nervous system aneurysm clips
6.	Implanted neural stimulator
7.	Any implanted device that is incompatible with MRI
8.	Unsatisfactory performance status as judged by the physician such that the participant could not tolerate an MRI scan
9.	Participants requiring monitored sedation for MRI studies
10.	Participants with a condition precluding entry into the scanner (for example, morbid obesity, claustrophobia)
11.	Participants with severe back-pain or motion disorders who will be unable to tolerate supine positioning within the MRI scanner and hold still for the duration of the examination
**Exclusion criteria for gadolinium-based MRI studies only:**	
1.	History of severe allergic reaction to gadolinium contrast agents despite pre-medication with diphenhydramine and prednisone
2.	Chronic kidney disease (estimated glomerular filtration rate of <60 mL/min/1.73 m^2^ of body surface area)

#### Plasma assays for markers of endothelial dysfunction and neurohormonal axis assessment

Markers of endothelial dysfunction and neurohormonal assays will be measured using plasma aliquoted from a single 10 mL blood draw collected at the indicated time points (Additional file 1: Figure S1). NT-proBNP testing will be performed on a separate blood sample sent to the NIH Clinical Center Laboratory. A timed 8-hour (overnight) urine sample will be collected, the total volume recorded, and 50 mL will be saved for further processing. Following centrifugation, urine sample aliquots will be stored at −80°C [51]. Plasma and urine aliquots for neurohormonal testing will be shipped to the University of Pennsylvania for further processing.

#### Gene expression profiling of peripheral blood mononuclear cells using Affymetrix GeneChip^®^ Microarrays

PBMCs have repeated contact with the altered endothelial cell surface in PAH and therefore may harbor unique signatures that are indicative of the disease process [52-54]. In a recent pilot study comparing PBMC expression profiles from 10 patients with PAH with 10 age-, gender- and race-matched controls, we identified 321 differentially regulated genes at a 20% false discovery rate. Ingenuity^®^ Pathway Analysis (Ingenuity^®^ Systems, Redwood, CA, USA) identified gene signatures for inflammation, cell-to-cell signaling and interaction, cytoskeletal rearrangement, cellular movement, hemostasis, and cell death [55]. To determine the effects of spironolactone therapy on PBMC gene expression in patients with PAH, we will compare changes in expression profiles at 12 and 24 weeks from baseline (obtained under our Natural History Study) between the treatment and placebo groups.

Blood specimens will be encoded to ensure patient confidentiality. No gene expression information with patient identifiers will be placed in the medical chart or in public records. Total RNA will be isolated, precipitated and quantified. Once total RNA is prepared, samples can be safely stored for an additional year. If longer storage of RNA samples is necessary, a commercially available methodology can be used to safely store total RNA at −80°C for up to 10 years. Total RNA isolated from the patient’s PBMCs will be hybridized to Affymetrix GeneChip^®^ Human Gene 2.0 ST Arrays (Affymetrix^®^, Santa Clara, CA, USA). Expression profile results will be used to direct confirmatory testing using quantitative real-time polymerase chain reaction (PCR).

#### Gene expression profiling of primary pulmonary artery endothelial cells exposed to plasma from participants with pulmonary arterial hypertension

Previous reports have used microarray analysis to PBMCs [52-54] and lung tissue [56,57] from patients with PAH, however a focused investigation of PAH plasma effects on primary human pulmonary artery endothelial cells (PAECs) has not been previously reported to our knowledge [58]. Plasma (from 60 mL of whole blood) will be collected from participants in sterile, endotoxin-free syringes containing heparin. Samples will be collected at baseline (under the Natural History Study) and upon study completion at 24 weeks. This plasma will be used to explore the effects of circulating mediators on PAEC gene expression. We have developed a bioassay assessing global transcriptomic changes in PAECs induced by plasma from patients with PAH compared to healthy controls using Affymetrix^®^ oligonucleotide microarrays. In addition to naive cells, plasma may be tested on PAECs exposed to inflammatory challenges and/or rendered ‘dysfunctional’ (bone morphogenetic protein receptor 2 gene silencing or Smad dominant negative mutant expression with or without endothelial nitric oxide synthase gene silencing). Candidate genes will be confirmed by quantitative real-time PCR. Gene expression changes will be correlated with pulmonary artery endothelial function assessed *in vivo* by MRI as well as with traditional measurements of disease severity, including NYHA/WHO class and 6-minute walk. Remaining stored plasma may later be tested for circulating factors such as microRNAs, cytokines, chemokines or other circulating mediators for correlation with expression profiling results.

### Statistical considerations and analysis of the study

#### Sample size

Previous studies have examined the effects of PAH-specific therapy on time to clinical worsening, exercise capacity and endothelial inflammation in patients with PAH [59-63]. Moreover, the effects of spironolactone on endothelial dysfunction have been examined in diverse non-PAH patient populations [19-23]. However, no prior randomized controlled trials have been completed that report on the effects of spironolactone treatment in patients with PAH. Assuming that a subset of the participants with PAH may discontinue study drug or otherwise not be able to complete the study, we plan to enroll up to 70 participants with PAH to obtain at least 50 completed studies. The participants will be randomized in a 1:1 ratio to spironolactone therapy or placebo. At a two-sided α level of 0.05, we will have at least 84% power to detect an effect size (group mean difference divided by standard deviation) of 0.9 for the difference in the change of 6-minute walk distance from baseline between the two treatment groups [61].

#### Study analysis

Participant characteristics will be summarized using contingency tables (for categorical variables), means and standard deviations for continuous variables that are approximately normally distributed (transformed if needed) or median and inter-quartile range for continuous variables that are not normal.

For the primary endpoints, changes in 6-minute walk distance (24 weeks versus baseline) will be compared between the two groups using linear mixed models (LMMs), Kaplan-Meier curves will be plotted to show the time to clinical worsening, and a log-rank test will be used to compare the two arms. Chi-squared tests or Fisher exact tests, t-tests or Wilcoxon rank-sum tests will be used to compare variables between the two arms when appropriate. The rates of study drug discontinuation between the two arms will be compared using the Fisher exact test, and a logistic regression will be considered to account for potential confounders if the two arms are imbalanced. The causes of discontinuation will be tabulated and compared if appropriate.

For secondary endpoints, LMMs will be used to assess the effect of spironolactone therapy on changes in VO_2_ maximum (24 weeks versus baseline), plasma markers of endothelial inflammation, sex hormone levels, and on activation of the renin-angiotensin-aldosterone and sympathetic nervous systems, as well as RV function as assessed by echocardiography, MRI and NT-proBNP. Random participant effect will be included to account for repeated measures within each participant over time. Transformations (for example, log-transformation) will be considered to stabilize variance. Standard residual diagnostics will be used to assess model assumption. VO_2_ maximum data may not be available for all participants. Based on the recent experience of the NHLBI pulmonary function laboratory at the NIH Clinical Center, approximately 5% to 10% of research participants have been unable to complete cardiopulmonary exercise testing or had incomplete data. We will collect information about the reason for missing VO_2_ maximum data. If the missing data can be considered ‘missing at random’ (for example, due to personal preference, orthopedic issues, inability to tolerate the mouthpiece or facemask), LMM is appropriate. For potentially informative missing data (for example, a participant is too sick to complete the procedure), to be conservative, a minimum value will be used for participants in the spironolactone group, and a last-observation-carry-forward approach will be used in the control group. The proportions of participants in the two treatment groups that have no VO_2_ maximum data will also be compared.

#### Microarray analysis

Samples and their corresponding results will be subjected to strict quality control standards to assure that specimens are processed in balanced batches, to minimize the lot and reagent batch effects. Raw data will be background corrected, transformed, quantile normalized and summarized using the robust multi-array average technique. A principal components analysis will be computed to assist with recognizing outlying microarrays, which, along with other quality control indicators, may lead to rejection or re-analysis of some samples. LMM will be used to compare the PBMC gene expression profiles between the two treatment groups at each time point after accounting for repeated measures of each patient. Gene selection will be based on calculated false discovery rate to control the number of false positive findings.

Significant genes from these analyses will be annotated using automated tools, and a literature search will be performed for relevant information relating to the set of significant results. Significantly over-represented terms from formal ontologies, such as GeneOntology using the Database for Annotation, Visualization and Integrated Discovery, will be reported. Additional bioinformatics tools such as, Ingenuity^®^ Pathway Analysis (Ingenuity^®^ Systems) will be used in the annotation step.

### Monitoring of participants and criteria for withdrawal from study

#### Spironolactone treatment

As a safeguard, at the time of enrollment each patient will be asked to identify a primary responsible physician who will be notified of the patient’s enrollment and, therefore, will be aware of the potential development of hyperkalemia or renal insufficiency during the course of the study. Serum potassium, blood urea nitrogen and creatinine will be measured one week after initiating therapy or a dose increase and then serially thereafter according to the pre-specified schedule (Figure 1 and Additional file 1: Figure S1). For laboratory monitoring of serum potassium, blood urea nitrogen and creatinine on day 7, week 6, week 9, week 16 and week 20 (±7 days), participants will be given laboratory requisition slips for sample collection at an outside facility near their home (Additional file 1: Figure S1). Participants will be instructed to have their samples drawn within 7 days of the specified time point. Those participants who wish to return to the NIH Clinical Center to have these samples drawn may do so instead of going to an outside facility. For laboratory monitoring of serum potassium, blood urea nitrogen and creatinine at week 12 and week 24, samples will be collected along with other research samples at the time of the NIH Clinical Center visit (Additional file 1: Figure S1). In the event that specimens are reported as hemolyzed, participants will be instructed to repeat their sample draw within 7 days of their previous sample. In the event that any scheduled laboratory monitoring is delayed for >7 days, participants will be instructed to stop the study drug and will be removed from the protocol.

**Figure 2:**
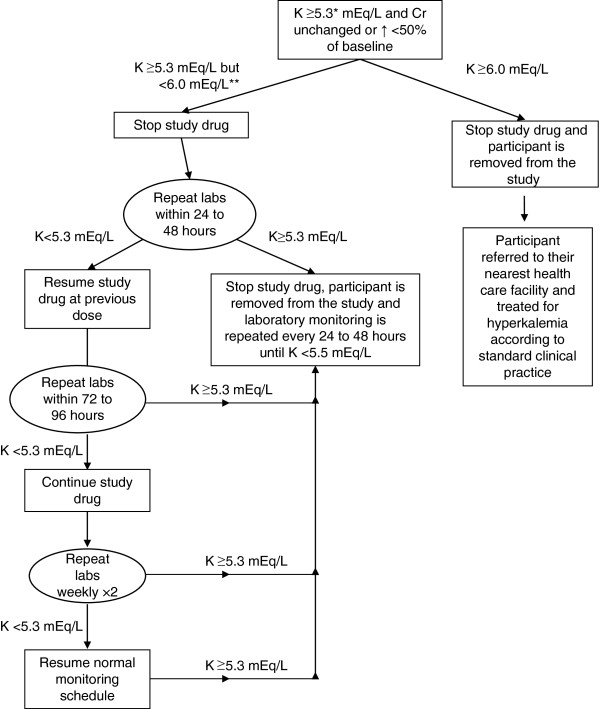
**Monitoring plan for participants who develop hyperkalemia.** *Throughout the study, participants with a potassium level >5.0 mEq/L will undergo dietary counseling regarding a low potassium diet. **At the investigators’ discretion, any participant with a potassium level ≥5.3 mEq/L can be referred for medical evaluation at the nearest health care facility. Cr, creatinine; K, potassium; labs, laboratory tests.

If while on therapy a patient’s serum potassium level increases to 5.3 to 5.9 mEq/L, the study drug will be stopped and their serum potassium level will be checked again within 24 to 48 hours (Figure 2). At the study investigators’ discretion, any participant with a potassium level ≥5.3 mEq/L can be referred for medical evaluation at the nearest health care facility. If the repeat serum potassium is <5.3 mEq/L, then the study drug will be resumed at the previous dose and serum potassium measurement will be repeated again 72 to 96 hours later, and then weekly for two weeks, followed by resumption of the normal monitoring schedule as long as the serum potassium remains <5.3 mEq/L. If the serum potassium remains ≥5.3 mEq/L on two consecutive blood draws, the study drug will be discontinued. Delay in laboratory monitoring longer than 48 hours from the specified time frame in participants with a serum potassium of ≥5.3 mEq/L will result in that patient being removed from the protocol. If at any time the serum potassium is ≥6.0 mEq/L (Figure 2), the study drug will be discontinued and the participant will be referred to the nearest health care facility where standard treatment for hyperkalemia can be provided. In these participants, the study drug will not be restarted and they will be removed from the protocol. If while on the study drug a participant’s serum creatinine increases >50% then the study drug will be discontinued and the participant will be removed from the protocol. Prior to initiation of the study drug and throughout the trial during follow-up visits, participants will be asked about the presence of breast pain and, in male participants, the development of gynecomastia.

#### Six-minute walk test

Throughout testing, all participants will be monitored by a respiratory therapist or a pulmonary function technician with pulse oximetry. The test will be terminated if the participant develops limiting clinical symptoms including but not limited to significant fatigue, shortness of breath, leg discomfort and dizziness.

#### Cardiopulmonary exercise testing

All participants will be accompanied by a pulmonary function technician and a licensed practitioner and will be monitored by continuous pulse oximetry and electrocardiography (ECG). Exercise testing will not be performed in participants with absolute contraindications to exercise testing according to American College of Cardiology/American Heart Association guidelines, which include the following: acute myocardial infraction (within 2 days), high-risk unstable angina, uncontrolled cardiac arrhythmias causing symptoms or hemodynamic compromise, symptomatic severe aortic stenosis, uncontrolled symptomatic heart failure, and acute aortic dissection. Determination of test termination will be based on published guidelines (Table 6) [64]. In addition, the test will be terminated regardless of symptoms if both of the following two conditions are met: pulse oximetry decreases by >5% from baseline and pulse oximetry value <90%.

**Table 6:** American College of Cardiology/American Heart Association indications for terminating exercise testing

**Absolute indications**	**Relative indications**
1. Decrease in systolic blood pressure >10 mmHg from baseline, accompanied by evidence of ischemia	1. Decrease in systolic blood pressure >10 mmHg from baseline, in the absence of evidence of ischemia
2. Moderate to severe angina	2. ST or QRS changes such as excessive ST-depression (>2 mm of horizontal or down-sloping ST-segment depression) or marked axis shift
3. Increasing nervous system symptoms (ataxia, dizziness, or near-syncope)	
4. Signs of poor perfusion (cyanosis or pallor)	3. Arrhythmias other than sustained ventricular tachycardia, including multifocal PVCs, triplets of PVCs, supraventricular tachycardia, heart block, or bradyarrhythmias
5. Technical difficulties in monitoring ECG or systolic blood pressure	
6. Participant’s desire to stop	4. Fatigue, shortness of breath, wheezing, leg cramps or claudication
7. Sustained ventricular tachycardia	5. Development of bundle-branch block or IVCD that cannot be distinguished from ventricular tachycardia
8. ST elevation (≥ 1.0 mm) in leads without diagnostic Q-waves (other than V_1_or aVR)	6. Increasing chest pain
	7. Hypertensive response^a^

^a^In the absence of definitive evidence, the committee suggests systolic blood pressure of >250 mmHg and/or a diastolic blood pressure of >115 mmHg. ECG, electrocardiography; IVCD, intraventricular conduction delay; PVCs, premature ventricular contractions.

#### Magnetic resonance imaging

MRI scans are expected to last 30 to 60 minutes but may get prolonged to 90 minutes in some cases. Scans are monitored by a technologist supervised by a physician or by a physician with the support of a second health care professional. Monitoring typically includes continuous ECG, intermittent non-invasive blood pressure, and pulse oximetry as indicated. Participants may elect to terminate the imaging protocol at any time.

MRI studies will be monitored in the following ways: direct visual and auditory contact with the participant by a nurse, nurse practitioner or physician in the MRI suite; indirect visual contact via a video camera output displayed over the MRI scan control console; two-way microphone system between the participant and the operator of the MRI scan control console; continuous display of the ECG; and intermittent non-invasive measurement of blood pressure.

### Individual participant stopping rules

PAH is a progressive disease and therefore clinical worsening may occur in participants during the protocol, irrespective of their treatment group assignment. Individual stopping rules for participants enrolled in the protocol include:

1. If a participant develops clinical evidence of right heart failure (which may include but is not limited to elevated jugular venous pressure, ascites and lower extremity edema) as determined by the patient’s primary physician or the investigators, then the participant will be removed from the protocol and treated for decompensated right heart failure according to standard of care.

2. If a participant deteriorates clinically, even in the absence of overt right heart failure, and the primary treating physician or the investigators feel that the addition of a MR antagonist such as spironolactone or eplerenone is clinically indicated then the participant will be removed from the study.

3. If a participant has more than one episode of serious hyperkalemia (potassium ≥5.3 mEq/L) or one episode of severe hyperkalemia (potassium ≥6.0 mEq/L), the study drug will be discontinued and the participant will be removed from the protocol.

4. If a participant develops renal insufficiency (rise in serum creatinine >50% of baseline) while on protocol, then the study drug will be discontinued and the participant will be removed from the protocol.

5. In the event that testing for serum potassium, blood urea nitrogen and creatinine is delayed for >7 days, the participant will be instructed to stop the study drug and will be removed from the protocol.

6. In any participant with a serum potassium level ≥ 5.3 mEq/L, a delay in laboratory monitoring longer than 48 hours from the specified time frame will result in removal from the protocol.

7. If while on study drug a participant develops significant breast pain or gynecomastia then the study drug will be discontinued and the participant will be removed from the protocol.

### Protocol stopping rules

Due to the fact that this protocol is designed as a pilot study of spironolactone therapy, we have not set predetermined protocol stopping rules for clinical efficacy. However, as described above, the occurrence of excess life-threatening or serious adverse events (such as severe hyperkalemia or renal insufficiency) is possible and the frequency and distribution of these events in each treatment group will be monitored closely. Serious adverse events and unexpected adverse events will be reviewed with the help of the Data Safety and Monitoring Board (DSMB) to determine the relationship of these events to the current protocol.

### Human participant protection

#### Rationale for participant selection

Criteria for exclusion or withdrawal from the study are based on the presence of other disease processes that may interfere with the interpretation of our results or situations that may be harmful to the participants. Individuals of both genders and all races will be considered for inclusion in this study. Cognitively impaired and institutionalized persons unable to provide written informed consent will not participate in this study.

#### Participation of children

Children under the age of 18 will be excluded from this protocol because of differences between adults and children with regards to functional classification and exercise capacity at the time of PAH diagnosis [65]. In addition, a substantially higher proportion of pediatric patients demonstrate acute vasoreactivity at the time of right heart catheterization compared to adults [65]. These differences would add additional heterogeneity to the study population and therefore could potentially obscure the effects of the study drug on exercise capacity, clinical worsening, RV function and vascular inflammation.

#### Participation of pregnant women

Because of potential adverse effects on the fetus, pregnant participants will be excluded from the study. In addition, women of childbearing potential must agree to use adequate contraception (hormonal, barrier method of birth control, or abstinence) prior to and for the duration of study participation. Female participants with childbearing potential will be regularly assessed throughout the study. In the event of a positive pregnancy test, the participant will be immediately informed of this result, the study drug will be discontinued, and the participant will be removed from the study.

### Risks and discomforts

#### Risks associated with spironolactone therapy

The most significant risks associated with spironolactone therapy include hyperkalemia, renal insufficiency, hypovolemia and gynecomastia with or without breast pain. In a large trial of patients with NYHA class III and IV LV heart failure (the mean dose of spironolactone was 26 mg and dose titration up to 50 mg daily), the incidence of severe hyperkalemia (>6 mEq/L) was not significantly different between the spironolactone treatment group (2%) and placebo (1%) but the median potassium concentration did increase by 0.30 mmol/L in the spironolactone-treated group [15]. The median creatinine concentration also increased (0.05 to 0.10 mg/dL) in the spironolactone-treated group. The risk of hyperkalemia while on spironolactone therapy is increased in the presence of renal insufficiency, concurrent use of ACE inhibitors or angiotensin receptor blockers, potassium-sparing diuretics or NSAIDs [66]. Precautions that will be taken include: participants will be counseled to avoid foods high in potassium and to avoid concurrent use of NSAIDs; patients taking ACE inhibitors, angiotensin receptor blockers, or potassium-sparing diuretics will be excluded from the study; participants will undergo frequent monitoring of serum potassium, creatinine and blood urea nitrogen; cessation of the study drug when serum potassium ≥6 mEq/L or when it remains ≥5.3 mEq/L on two occasions; and cessation of the study drug when creatinine increases by >50% of baseline value.

Gynecomastia in men and painful breast enlargement in women may occur in participants treated with spironolactone and appear to be related to dose and duration of therapy [67-69]. The Randomized Aldactone Evaluation Study reported incidences of gynecomastia and breast pain in 9% and 2% of spironolactone-treated patients, respectively. This was significantly different from placebo and led to discontinuation of the study drug in 2% of spironolactone-treated patients in this trial [15]. In the majority of cases, gynecomastia and breast pain subside with cessation of the drug [68,70]. Prior to initiation of the study drug and throughout the trial during follow-up visits, participants will be asked about the presence of breast pain and, in male patients, the development of gynecomastia. If while on the study drug a participant develops significant breast pain or gynecomastia then the study drug will be discontinued. Additional side effects reported with spironolactone therapy and related to its progesteronal and anti-androgenic activity include menstrual irregularities (amenorrhea, postmenopausal bleeding), decreased libido and erectile dysfunction [67-69]. These also appear to be dose related and typically resolve upon drug cessation [69,71].

#### Risks associated with venipuncture

Blood draws throughout the study are well within the NIH Clinical Center policy (that is, the amount of blood drawn for research purposes from participants 18 years of age or older will not exceed 550 mL or 10.5 mL/kg, whichever is smaller, over any eight-week period). Standard precautions for obtaining human blood samples will be taken. Transient discomfort and minor bruising may occur at the phlebotomy site. Vasovagal symptoms can occur during blood drawing.

#### Six-minute walk test

The risks associated with a symptom-limited six-minute walk are minimal.

#### Cardiopulmonary exercise testing

Exercise testing is a safe procedure with experienced personnel. Conduction abnormalities or atrial/ventricular arrhythmias may occur during the test, requiring institution of advanced cardiovascular life support. The participant could also develop ischemic symptoms requiring intensive care unit monitoring and treatment.

#### Transthoracic echocardiography

There is no known risk from the ultrasound examination. The safety of echocardiography has been well established. Localized transient discomfort from the probe and irritation from the ultrasound gel may occur.

#### Magnetic resonance imaging and gadolinium contrast agent

MRI uses no ionizing radiation and is quite safe when performed on a properly screened population. Potential risks relate to the magnetic field’s effect on participants with implanted metal objects (such as cerebral aneurysm clips, cochlear implants). The magnetic field can cause twisting or movement of these objects thus causing harm to the patient. Also the radiofrequency power associated with MRI can potentially cause burns in patients with pacemakers or other implanted coiled wires. Participants will be screened for these objects and excluded as indicated. The scanner gradient performance (dB/dt limit) is set on an individual basis to avoid painful peripheral nerve stimulation. Precautions that will be taken include informing the participant of the possibility for peripheral nerve stimulation in the consent form; empirically using parameters that maintain gradient switching rates below the pain threshold; and audio feedback from the participant.

The NIH MRI Unit adheres to the Food and Drug Administration guidelines for radiofrequency power levels (specific absorption rate limits). The MRI scanner is operated at the Food and Drug Administration limits established for MRI in general applications. However, in cases of a damaged or dysfunctional surface coil, there is a small chance of local warming in the body. For safety purposes, participants are asked to report this sensation so scans can be modified or the integrity of hardware verified.

Participants may receive an intravenous injection of gadolinium chelate that is not to exceed 0.2 mmol/kg gadolinium per bolus injection and 0.4 mmol/kg gadolinium per examination. We will be adhering to the NHLBI polices regarding administration of gadolinium. Experience with a large number of patients has shown that these commercially available gadolinium chelates are safe [72-76] and without side effects in the majority (>98%) of patients. When side effects do occur, they are usually mild and transient. These include coldness in the arm during the injection, a metallic taste, headache and nausea. More severe reactions (shortness of breath, wheezing or hypotension) are extremely rare. More recently however, there have been reports of nephrogenic systemic fibrosis or nephrogenic fibrosing dermopathy associated with the use of gadolinium in patients with severe renal dysfunction [77]. The risk of these occurrences should be minimized through the use of a questionnaire form and an assessment of eGFR in all participants.

### Data and safety monitoring

#### Clinical monitoring plan

The principal investigator will monitor accrual and safety data as well as occurrence of any unusual or unpredicted complications. An independent DSMB established by the NHLBI will review symptom and outcome data at 6-month intervals to ensure participant safety and confirm that withdrawal criteria are observed. Study monitoring will be conducted by an outside consultant working under an agreement with the NHLBI to monitor aspects of the study in accordance with the appropriate regulations and the approved protocol. Stopping rules and withdrawal criteria have been established to ensure participant safety during the study. The DSMB may recommend early termination of the study for considerations of safety and efficacy. Accrual and safety data will also be monitored and reviewed annually by the Institutional Review Board.

#### Data management

The principal investigator will be responsible for overseeing entry of data into a password-protected electronic system and ensuring data accuracy, consistency and timeliness. On all study documents, participants will be identified by a participant number assigned at enrollment, and will not be identified by name. Data will be stored in locked cabinets and in a password-protected database until it is no longer of scientific value. Should we become aware that a major breech in our plan to protect patient confidentiality and trial data has occurred, the Institutional Review Board will be notified.

#### Adverse events and serious adverse events reporting

Adverse events reported under this protocol will be limited to those events that are possibly, probably or definitely related to the research described in this protocol.

Unanticipated problems that are either adverse events or non-adverse events will be reported within 7 calendar days of investigator awareness. The following items will also be reported to the NHLBI Institutional Review Board in summary at the time of Continuing Review: all unanticipated problems; any protocol-specific reporting requirements; and all protocol deviations, which in the opinion of the investigator require reporting.

## Discussion

Currently no published data exists from randomized clinical trials examining the safety and efficacy of MR antagonist therapy in early stages of PAH. Initiating therapy with spironolactone at an earlier stage of disease may improve PAH-associated vascular inflammation. This protocol describes the rationale, design and methodology of a randomized, double blinded, placebo-controlled pilot study in patients with PAH investigating the effect of early treatment with spironolactone on exercise capacity, clinical worsening and vascular inflammation *in vivo*.

### Trial status

We are in the recruiting phase.

## Abbreviations

ACE: angiotensin-converting enzyme; Cr: creatinine; DSMB: Data Safety and Monitoring Board; ECG: electrocardiography; eGFR: estimate glomerular filtration rate; HIV: human immunodeficiency virus; IL-1β: interleukin-1 beta; IL1RL1: interleukin-1 receptor-like 1; IL-6: interleukin-6; IL-7: interleukin-7; IL-8: interleukin-8; IPAH: idiopathic pulmonary arterial hypertension; IVCD: intraventricular conduction delay; K: potassium; labs: laboratory tests; LMM: linear mixed model; LV: left ventricular; MCP-1: monocyte chemotactic protein-1; MET: metabolic equivalent, MPH, miles per hour; MR: mineralocorticoid receptor; MRI: magnetic resonance imaging; NFκB: nuclear factor kappa B; NHLBI: National Heart, Lung, and Blood Institute; NIH: National Institutes of Health; NR: nuclear receptor; NSAIDs: nonsteroidal anti-inflammatory drugs; NT-proBNP: N-terminal pro-brain natriuretic peptide; NYHA: New York Heart Association; PAECs: pulmonary artery endothelial cells; PAH: pulmonary arterial hypertension; PBMCs: peripheral blood mononuclear cells; PCR: polymerase chain reaction; PH: pulmonary hypertension; PVCs: premature ventricular contractions; RNA: ribonucleic acid; RV: right ventricular; sCD40L: soluble CD40 ligand; sICAM-1: soluble intercellular adhesion molecule 1; sVCAM-1: soluble vascular cell adhesion molecule 1; TNFα: tumor necrosis factor alpha; VCO2: carbon dioxide production; VE: ventilation; VO2: oxygen uptake; WHO: World Health Organization.

## Competing interests

The authors declare that they have no competing interests.

## Authors’ contributions

JME, RLD and MAS contributed to the conception and design of the protocol and drafted the initial manuscript. JER, PRF, MKH, JS, AG, KA, GG, and BH made contributions to the conception and design of the trial and critically revised the manuscript for important intellectual content. All authors read and approved of the final manuscript.

## Supplementary Material

Additional file 1: Figure S1 Time and events schedule. Click here for file
