# Supplementary material for: A pilot study of the effect of spironolactone therapy on exercise capacity and endothelial dysfunction in pulmonary arterial hypertension: study protocol for a randomized controlled trial
Source: Trials. 2013 Apr 2;14:91. doi: 10.1186/1745-6215-14-91 (PMC3653687; doi:10.1186/1745-6215-14-91)
Supplement: Additional file 1: Figure S1 — Time and events schedule. [file 1745-6215-14-91-S1.docx]

**Figure S1. Time and events schedule**

Time points shaded in orange correspond to NIH Clinical Center visits to be performed under the companion Natural History Study and shared with this study. Time points shaded in green correspond to NIH Clinical Center visits under this protocol. Time points shaded in gray correspond to lab testing done at an outside facility. The 8-week time point (shaded in blue) corresponds to the approximate timing of study drug dose increase in participants meeting pre-specified criteria. *Obtained only in participants with a known history of HIV infection who have not had more than one viral load test done in the past 12 months. **If clinically indicated.

| **Procedure** | **Day -3 to -1**  **(Performed under**  **Natural History Study)** | **Day 0-3** | **Day**  **7** | **Week**  **6** | **Week**  **8** | **Week**  **9** | **Week 12** | **Week 16** | **Week 20** | **Week 24** |
| --- | --- | --- | --- | --- | --- | --- | --- | --- | --- | --- |
| History and physical | **X** |  |  |  |  |  |  |  |  |  |
| Clinic visit |  |  |  |  |  |  | **X** |  |  | **X** |
| CBC with differential and PT/PTT | **X** |  |  |  |  |  | **X** |  |  | **X** |
| Basic metabolic and hepatic panel | **X** |  |  |  |  |  | **X** |  |  | **X** |
| HIV viral load* | **X** |  |  |  |  |  |  |  |  |  |
| ß-hcg for women of child bearing potential | **X** |  |  |  |  |  | **X** |  |  | **X** |
| Electrocardiogram | **X** |  |  |  |  |  |  |  |  |  |
| Basic metabolic panel (including potassium, creatinine and blood urea nitrogen) |  |  | **X** | **X** |  | **X** |  | **X** | **X** |  |
| NYHA/WHO functional class assessment | **X** |  |  |  |  |  | **X** |  |  | **X** |
| Right heart catheterization** | **X** |  |  |  |  |  |  |  |  |  |
| Echocardiogram | **X** |  |  |  |  |  | **X** |  |  | **X** |
| Magnetic resonance imaging | **X** |  |  |  |  |  | **X** |  |  | **X** |
| Six minute walk testing | **X** |  |  |  |  |  | **X** |  |  | **X** |
| Cardiopulmonary exercise testing | **X** |  |  |  |  |  |  |  |  | **X** |
| Plasma collection for markers of endothelial cell inflammation | **X** |  |  |  |  |  | **X** |  |  | **X** |
| Plasma collection for markers of neurohormonal activation and soluble ST2 |  | **X** |  |  |  |  | **X** |  |  | **X** |
| Plasma NT-proBNP | **X** |  |  |  |  |  | **X** |  |  | **X** |
| Urine collection for steroid metabolites |  | **X** |  |  |  |  | **X** |  |  | **X** |
| Blood collection for PBMC gene expression profiling | **X** |  |  |  |  |  | **X** |  |  | **X** |
| Plasma collection for *in vitro* pulmonary artery endothelial cell stimulation | **X** |  |  |  |  |  |  |  |  | **X** |
